# Association Between User Interaction and Treatment Response of a Voice-Based Coach for Treating Depression and Anxiety: Secondary Analysis of a Pilot Randomized Controlled Trial

**DOI:** 10.2196/49715

**Published:** 2023-11-06

**Authors:** Nan Lv, Thomas Kannampallil, Lan Xiao, Corina R Ronneberg, Vikas Kumar, Nancy E Wittels, Olusola A Ajilore, Joshua M Smyth, Jun Ma

**Affiliations:** 1 Department of Medicine University of Illinois Chicago Chicago, IL United States; 2 Department of Anesthesiology Washington University St. Louis, MO United States; 3 Institute for Informatics Washington University St. Louis, MO United States; 4 Department of Epidemiology and Population Health Stanford University Stanford, CA United States; 5 Department of Psychiatry University of Illinois Chicago Chicago, IL United States; 6 Department of Psychology The Ohio State University Columbus, OH United States

**Keywords:** user interaction, treatment alliance, treatment response, voice assistant, depression, anxiety

## Abstract

**Background:**

The quality of user interaction with therapeutic tools has been positively associated with treatment response; however, no studies have investigated these relationships for voice-based digital tools.

**Objective:**

This study evaluated the relationships between objective and subjective user interaction measures as well as treatment response on Lumen, a novel voice-based coach, delivering problem-solving treatment to patients with mild to moderate depression or anxiety or both.

**Methods:**

In a pilot trial, 42 adults with clinically significant depression (Patient Health Questionnaire-9 [PHQ-9]) or anxiety (7-item Generalized Anxiety Disorder Scale [GAD-7]) symptoms or both received Lumen, a voice-based coach delivering 8 problem-solving treatment sessions. Objective (number of conversational breakdowns, ie, instances where a participant’s voice input could not be interpreted by Lumen) and subjective user interaction measures (task-related workload, user experience, and treatment alliance) were obtained for each session. Changes in PHQ-9 and GAD-7 scores at each ensuing session after session 1 measured the treatment response.

**Results:**

Participants were 38.9 (SD 12.9) years old, 28 (67%) were women, 8 (19%) were Black, 12 (29%) were Latino, 5 (12%) were Asian, and 28 (67%) had a high school or college education. Mean (SD) across sessions showed breakdowns (mean 6.5, SD 4.4 to mean 2.3, SD 1.8) decreasing over sessions, favorable task-related workload (mean 14.5, SD 5.6 to mean 17.6, SD 5.6) decreasing over sessions, neutral-to-positive user experience (mean 0.5, SD 1.4 to mean 1.1, SD 1.3), and high treatment alliance (mean 5.0, SD 1.4 to mean 5.3, SD 0.9). PHQ-9 (*P*_trend_=.001) and GAD-7 scores (*P*_trend_=.01) improved significantly over sessions. Treatment alliance correlated with improvements in PHQ-9 (Pearson *r*=–0.02 to –0.46) and GAD-7 (*r*=0.03 to –0.57) scores across sessions, whereas breakdowns and task-related workload did not. Mixed models showed that participants with higher individual mean treatment alliance had greater improvements in PHQ-9 (β=–1.13, 95% CI –2.16 to –0.10) and GAD-7 (β=–1.17, 95% CI –2.13 to –0.20) scores.

**Conclusions:**

The participants had fewer conversational breakdowns and largely favorable user interactions with Lumen across sessions. Conversational breakdowns were not associated with subjective user interaction measures or treatment responses, highlighting how participants adapted and effectively used Lumen. Individuals experiencing higher treatment alliance had greater improvements in depression and anxiety. Understanding treatment alliance can provide insights on improving treatment response for this new delivery modality, which provides accessibility, flexibility, comfort with disclosure, and cost-related advantages compared to conventional psychotherapy.

**Trial Registration:**

ClinicalTrials.gov NCT04524104; https://clinicaltrials.gov/study/NCT04524104

## Introduction

In 2020, nearly 1 in 5 US adults (~52 million) lived with a mental illness, and more than half (53.8%) of them did not receive any mental health services for psychotherapy or pharmacotherapy in outpatient or inpatient settings in the past year [1]. Reasons for this treatment gap included fears of stigmatization and access barriers due to cost, low reimbursement, service unavailability, or geography [2,3]. This lack of needed mental health care is especially acute among racial and ethnic minorities [4].

Evidence-based psychotherapies using conventional delivery modalities are many [5]; however, their reach and adoption in mental health or general medical settings are limited. As such, there is a critically unmet need for empirically validated psychotherapies that are low cost, avoid stigma, and can be delivered in an on-demand manner to help address the growing public health and health equity challenges.

Digital mental health interventions have shown considerable potential to address the particular issues of reach and access [6,7]. However, studies on their effectiveness, user engagement, and prolonged use have produced mixed results [7,8]. For example, some of these interventions have been shown to be as effective as traditional psychotherapy and pharmacotherapy in improving depression and anxiety [9,10], whereas the effectiveness of others has remained inconclusive [11]. In addition, participant adherence to digital interventions varies largely, with estimates ranging from 6% to 100%, with lower adherence in practice than in research trials [12].

One of the key determinants in the success of digital mental health interventions is the ability to conduct streamlined user interactions [13]. Assessing interactions in digital mental health interventions is paramount for optimizing treatment adherence and outcomes. Measures of user interactions, including objective measures such as the frequency of breakdowns during user interaction with a digital intervention, and subjective measures such as participant-reported task-related workload [14], usability [15], and treatment alliance for digital interventions [16], can provide insights on the pragmatic and translational use of these interventions. However, research on the relationship between user interactions and treatment outcomes of digital mental health interventions is scarce [8].

A new class of digital mental health interventions includes voice-based artificial intelligence (AI) coaches that have shown potential for delivering personalized and accessible mental health therapy [17]. Such voice-based coaches can be developed on consumer-based voice assistant platforms (eg, Amazon’s Alexa or Google Home) to deliver therapy. Being a new therapeutic delivery form, the understanding of its voice-based user interactions for treatment and associations with patient outcomes is lacking. With known challenges such as natural language understanding with voice assistants [17,18], conversational breakdowns can occur where the device platform (eg, Alexa) cannot properly recognize a participant’s voice input. It is unknown, however, whether such breakdowns affect participants’ subjective assessment of their interactions, their perceived alliance with the treatment delivered, or their treatment outcomes.

In this secondary analysis of a recently completed pilot randomized clinical trial (RCT) [19], we evaluated the relationships between objective and subjective user interaction measures as well as treatment response on Lumen, a novel voice-based coach, delivering problem-solving treatment (PST) to patients with mild to moderate depression or anxiety or both.

## Methods

### Participants

Participants were recruited between April 5 and October 7, 2021, from the outpatient care clinics at the University of Illinois Hospital and Health Sciences System and employee email listserve (L-Soft International, Inc) at the University of Illinois Chicago (UIC), a minority-serving institution. The study was registered on ClinicalTrials.gov (NCT04524104). Enrolled participants had a 9-item Patient Health Questionnaire-9 (PHQ-9) score of 10-19 or a 7-item Generalized Anxiety Disorder Scale (GAD-7) score of 10-14 or both, without serious medical or psychiatric comorbidities or other exclusions [19]. A total of 63 participants were randomly assigned in a 2:1 ratio to receive the Lumen intervention (n=42) or to be in a waitlist control group (n=21). The pilot RCT demonstrated decreased depression and anxiety symptoms in the Lumen intervention group compared with the control group [19]. This study analyzed participant data only within the Lumen intervention group.

### Lumen Intervention

Lumen is a voice-based coach, developed on Amazon’s Alexa platform. Lumen delivers an evidence-based PST program [17,20,21] consisting of 8 sessions (4 weekly sessions and then 4 biweekly sessions over 12 weeks) for patients with mild to moderate depression or anxiety or both. PST is a participant-driven behavioral therapy, where the coach guides participants to identify a problem, set a goal, brainstorm solutions, choose a solution, develop an action plan, and implement and evaluate the plan [22]. An uninterrupted Lumen session lasted ~12 minutes.

Lumen was integrated into the Alexa app on an iPad. Participants using Lumen were longitudinally monitored via surveys delivered via text messages, integrated with a Research Electronic Data Capture (REDCap) database.

### User Interaction and Response Measures

Objective and subjective measures of user interaction included the number of voice-based conversational breakdowns during each session and self-administered surveys of workload, user experience, and the treatment alliance between the participant and Lumen after each session.

A conversational breakdown was defined as instances where a participant’s voice input or response could not be interpreted by Lumen. Such conversational breakdowns resulted in the participant having to repeat or correct their response to move on to the next part of their coaching session. Conversational breakdowns could occur due to a variety of reasons including incorrect invocation (ie, a participant says an incorrect phrase), incomplete invocation (ie, a participant says an incomplete phrase in response to Lumen), incomprehensible invocation (ie, a participant says something Alexa could not understand), repeated invocation (ie, a participant repeats the same answer multiple times), and internet issues (ie, the participant has network issues leading to his or her voice input not being received). For ascertaining such conversational breakdowns with Lumen on the Alexa platform, we extracted participant conversations with the Lumen Alexa skill in a text format and coded all the instances of breakdowns (based on the aforementioned reasons) and computed counts of such breakdowns per user session.

Workload was measured with a modified version of the National Aeronautics and Space Administration (NASA) Task Load Index (TLX) [14]. The TLX rating sheet was administered assuming similar weights for each of the 5 task load items: mental demand, temporal demand (eg, being rushed), effort, frustration, and performance. The original TLX includes a physical demand item which was not included herein, as it was not applicable for the task of interacting with Lumen. An overall TLX score was calculated as the sum of the 5 task load items, each ranging from 1 to 7. A higher overall score reflected greater (unfavorable) demand.

The user experience was measured with the 10-item User Experience Questionnaire Short Version (UEQ-S) [15]. From the UEQ-S survey, scale values were calculated by rescaling the survey responses to the range of –3 to 3 and the UEQ-S total score was calculated as the mean of survey responses. The UEQ-S total scores of <–0.8 represented a negative evaluation, between –0.8 and 0.8 represented a neutral evaluation, and >0.8 represented a positive evaluation [23].

The treatment alliance was measured with the 36-item Working Alliance Inventory-Technology Version (WAI-Tech) [16]. WAI-Tech is an adapted measure to measure treatment alliance with digital interventions. From the WAI-Tech survey, an overall score was calculated based on item mean. A higher overall score reflected a greater treatment alliance.

A total of 2 response measures—PHQ-9 and GAD-7—were self-reported before each Lumen session. The PHQ-9 measures depression symptoms, with a score ranging between 0 (best) and 27 (worst) [24]. The GAD-7 measures anxiety symptoms, with a score ranging between 0 (best) and 21 (worst) [25].

### Statistical Analysis

Descriptive summaries were generated for participant baseline characteristics, and user interaction and response measures for each session. The Pearson correlations between each possible pair of the 4 user interaction measures at each session for 8 sessions were obtained. The Pearson correlations between each user interaction measure for a session (eg, session 1) and a response measure completed before the immediate next session (eg, PHQ-9 or GAD-7 change at session 2 from session 1) also were obtained. Given the exploratory nature, we opted to not adjust for multiple comparisons in accordance with statistical and publication guidelines [26]. Instead, we focus on the strength (eg, moderate or stronger correlation *r*≥0.4 [27]) and pattern (eg, consistency across sessions) of associations in our data interpretation.

Given the expected relationship between treatment alliance and response, we performed mixed models to evaluate whether the participants’ reported treatment alliance with Lumen predicted their treatment response across the intervention sessions. Each participant’s treatment alliance was coded as 2 variables: the person mean of total sessions and the deviation of individual sessions from the person mean. The response outcomes were PHQ-9 and GAD-7 score changes from session 1, which were analyzed in separate models. The fixed effects of each model included the 2 treatment alliance variables and the number of total sessions completed by the time of response outcome data collected, adjusting for PHQ-9 or GAD-7 score at session 1, sex, race or ethnicity, education, and digital health literacy score. The random effect accounted for repeated measures with an autoregressive covariance matrix.

### Ethical Considerations

The UIC Institutional Review Board approved the study (STUDY2020-0918). All participants provided written informed consent.

## Results

### Subject Characteristics

Table 1 shows the mean values for baseline characteristics. The 42 intervention participants had a mean age of 38.9 (SD 12.9) years, 28 (67%) were women, 8 (19%) were Black, 12 (29%) were Latino, 28 (67%) had a high school or college (1 to 4 or more years) education, and 19 (45%) had an annual income less than US $55,000. On average, the participants had moderate depression (mean PHQ-9 score 12.7, SD 3.0) and anxiety (mean GAD-7 score 9.8, SD 2.5).

**Table 1:** Baseline characteristics.

Characteristic		Intervention (n=42)
Age (years), mean (SD)		38.9 (12.9)
Female, n (%)		28 (66.7)
**Race or ethnicity, n (%)**		
	Non-Hispanic White	15 (35.7)
	African American	8 (19.1)
	Asian or Pacific Islander	5 (11.9)
	Hispanic	12 (28.6)
	Other (eg, decline to state and multirace)	2 (4.7)
**Education, n (%)**		
	High school or GED^a^ or less	1 (2.4)
	College—1 year to 3 years	10 (23.8)
	College—4 years or more	17 (40.5)
	Postcollege education	14 (33.3)
**Annual family income (US $), n (%)**		
	<35,000	9 (21.4)
	35,000-<55,000	10 (23.8)
	55,000-<75,000	6 (14.3)
	≥75,000	17 (40.5)
**Digital health literacy, n (%)**		
	Low 1-1.999	0 (0.0)
	Medium 2-2.999	7 (16.7)
	High 3-4	35 (83.3)
PHQ-9^b^ score, mean (SD)		12.7 (3.0)
GAD-7^c^ score, mean (SD)		9.8 (2.5)

^a^GED: General Educational Development.

^b^PHQ-9: Patient Health Questionnaire-9.

^c^GAD-7: 7-item Generalized Anxiety Disorder Scale.

### User Interaction and Response

Table 2 shows the mean values for user interaction and response measures across the 8 intervention sessions. Mean session conversational breakdowns ranged 2.3 (SD 1.8) to 6.5 (SD 4.4) and showed a decreasing trend across sessions. The mean overall task-related workload ranged 14.5 (SD 5.6) to 17.6 (SD 5.6) out of a total possible score of 35; the task-related workload increased for session 2, but then decreased over the next 6 sessions.

**Table 2:** User interaction and treatment response measures by intervention session^a^.

				Session^a^													
				S1		S2		S3		S4	S5		S6		S7		S8
**User interaction measures**																	
	**Breakdown count**																
		Participants, n	38		37		37		37		36	35		34		34	
		Mean (SD)	4.7 (4.9)		6.5 (4.4)		4.3 (3.4)		3.6 (3.1)		4.4 (3.9)	3.1 (2.0)		2.3 (1.8)		2.9 (2.5)	
	**NASA TLX^b^**																
		Participants, n	33		29		24		24		22	27		23		27	
		Mean (SD)	14.5 (5.6)		17.6 (5.6)		17.1 (5.1)		17.0 (6.4)		17.4 (5.1)	16.4 (5.6)		15.1 (4.1)		16.0 (5.9)	
	**UEQ-S^c^**																
		Participants, n	33		29		24		24		22	27		23		27	
		Mean (SD)	1.1 (0.9)		0.8 (1.2)		0.5 (1.4)		0.6 (1.4)		0.7 (1.5)	0.6 (1.5)		1.0 (1.2)		1.1 (1.3)	
	**WAI-Tech^d^**																
		Participants, n	33		29		24		24		22	27		23		27	
		Mean (SD)	5.3 (0.9)		5.3 (0.9)		5.1 (1.1)		5.1 (1.2)		5.0 (1.4)	5.1 (1.1)		5.1 (1.2)		5.2 (1.1)	
**Treatment response measures**																	
	**PHQ-9 scores^e^**																
		Participants, n	38		38		38		37		37	35		34		34	
		Mean (SD)	10.3 (5.2)		9.4 (4.5)		8.6 (5.7)		8.2 (5.8)		7.7 (5.4)	7.1 (5.5)		7.3 (6.2)		6.9 (6.1)	
	**GAD-7 scores^f^**																
		Participants, n	38		38		38		37		37	35		34		34	
		Mean (SD)	9.4 (4.1)		8.4 (3.9)		7.8 (4.4)		7.0 (4.3)		6.5 (4.6)	6.5 (4.3)		7.0 (5.4)		6.6 (4.7)	

^a^S1-S8: Session 1 to Session 8.

^b^NASA TLX: National Aeronautics and Space Administration Task Load Index. An overall task load index score was calculated as sum of 5 task load items: mental demand, temporal demand (eg, being rushed), effort, frustration and performance, each ranging 1 to 7. A higher scores reflected unfavorable greater demand.

^c^UEQ-S: User Experience Questionnaire Short Version. Survey responses were rescaled to the range –3 to 3 and UEQ-S total score were calculated as mean of survey responses. Total score values <–0.8 represent a negative evaluation, between –0.8 and 0.8 represent a neutral evaluation, and >0.8 represent a positive evaluation on each scale.

^d^WAI-Tech: Working Alliance Inventory-Technology Version. An overall score was calculated as item mean, ranging 1 to 7. A higher overall score reflected a more positive rating of working alliance.

^e^PHQ-9: Patient Health Questionnaire-9. PHQ-9 scores range from 0 to 27 with higher scores indicating more severe depressive symptoms.

^f^GAD-7: Generalized Anxiety Disorder-7. GAD-7 scores range from 0 to 21 with higher scores representing more severe levels of anxiety.

Participants had a positive overall evaluation (UEQ-S total score values>0.8) of their user experience with Lumen for sessions 1, 2, 7, and 8 (mean 0.8, SD 1.2 to mean 1.1, SD 1.3) and a neutral overall evaluation (–0.8≤values≤0.8) for sessions 3-6 (mean 0.5, SD 1.4 to mean 0.7, SD 1.5).

The overall scores on the 7-point WAI-tech survey (mean 5.0, SD 1.4 to mean 5.3, SD 0.9) were moderately stable and high across sessions, indicating that Lumen-based PST sessions were perceived to align with the participants’ therapeutic needs, address their treatment goals, and have a high degree of liking and attachment.

**Figure 1:**
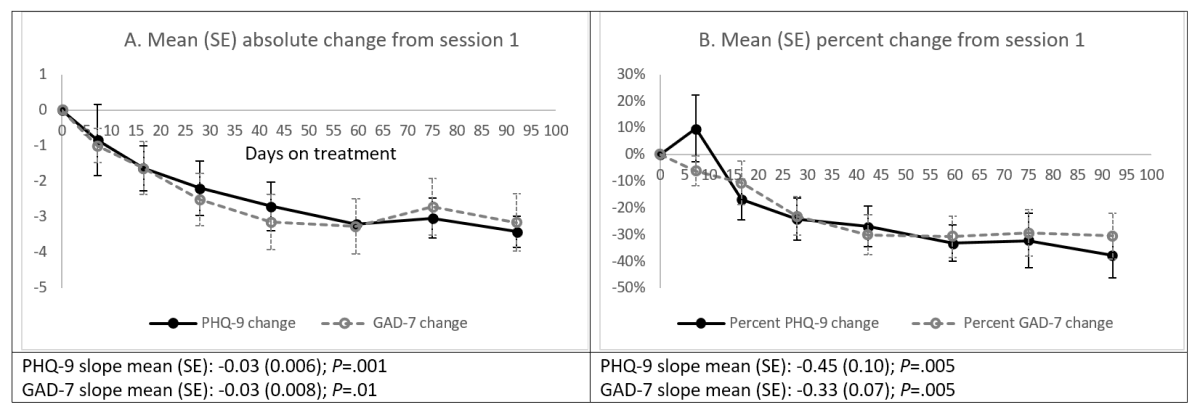
Trends of changes in Patient Health Questionnaire-9 and 7-item Generalized Anxiety Disorder Scale scores from session 1. Error bars indicate SE. GAD-7: 7-item Generalized Anxiety Disorder Scale; PHQ-9: Patient Health Questionnaire-9.

Figure 1 shows trends of absolute and percent PHQ-9 and GAD-7 changes from session 1. Both PHQ-9 (*P*_trend_=.001) and GAD-7 scores (*P*_trend_=.01) improved significantly over time, decreasing from a mean (SD) of 10.3 (SD 5.2) and 9.4 (SD 4.1) at session 1 to 6.9 (SD 6.1) and 6.6 (SD 4.7) at session 8. By session 8, participants had a 3.4 (SD 4.8) decline in PHQ-9 scores and a 3.2 (SD 4.7) decline in GAD-7 scores from session 1, which are equivalent to 37.8% (SD 49.3%) decline in PHQ-9 and 30.5% (SD 49.3%) decline in GAD-7.

### Correlations Between User Interaction Measures

**Figure 2:**
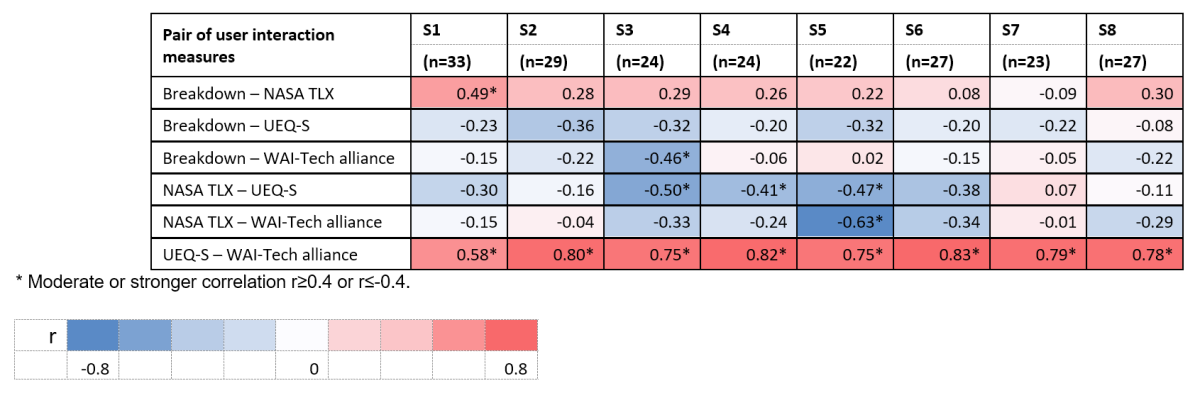
Correlations among user interaction measures by intervention session. Bivariate Pearson correlations were conducted. Each row provides the bivariate Pearson’s correlation coefficients between a pair of user interaction measures across 8 intervention sessions. NASA TLX: National Aeronautics and Space Administration Task Load Index; S1-S8: Session 1-Session 8; UEQ-S: User Experience Questionnaire Short Version; WAI-Tech: Working Alliance Inventory-Technology Version.

Figure 2 shows bivariate correlations among user interaction measures by intervention session. Conversational breakdowns were not moderately or strongly correlated with user experience across all 8 sessions or with overall task-related workload and treatment alliance for 7 out of 8 PST sessions (all *r*<0.40). User experience was positively correlated with treatment alliance across all 8 sessions (*r*=0.58-0.83, all *P*<.001).

### Correlations Between User Interaction Measures and Treatment Response

**Figure 3:**
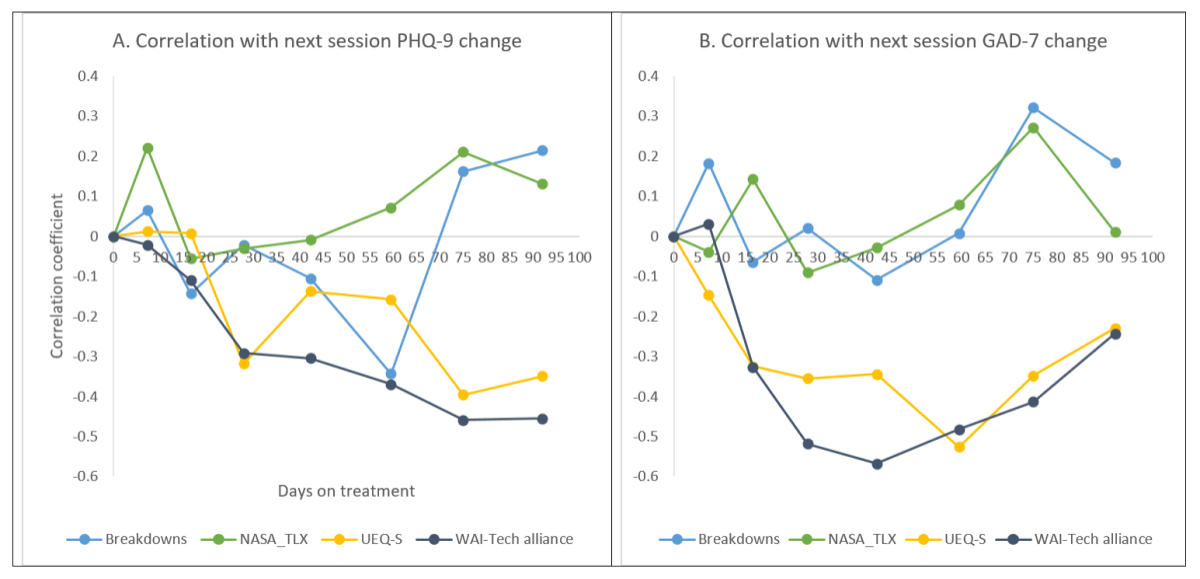
Correlations of user interaction with changes in next-session Patient Health Questionnaire-9 and 7-item Generalized Anxiety Disorder Scale scores from session 1. Bivariate Pearson correlations were conducted. GAD-7: 7-item Generalized Anxiety Disorder Scale; NASA TLX: National Aeronautics and Space Administration Task Load Index; PHQ-9: Patient Health Questionnaire-9; UEQ-S: User Experience Questionnaire Short Version; WAI-Tech: Working Alliance Inventory-Technology Version.

Figure 3 and Multimedia Appendix 1 show bivariate correlations of user interaction measures with next session PHQ-9 and GAD-7 changes from session 1. The number of conversational breakdowns and overall task-related workload of a session was not moderately or strongly correlated with PHQ-9 and GAD-7 score changes at the next session (all *r*<0.40). However, user experience at a session correlated with the ensuing session PHQ-9 (*r*=0.01 to –0.40) and GAD-7 (*r*=–0.15 to –0.53) changes from session 1. Most of these correlations were negative indicating that better user experience at the previous session (eg, session 3) was associated with a greater decline (improvement from session 1) in either PHQ-9 or GAD-7 at the next session (eg, session 4). Moreover, treatment alliance at the previous session (eg, session 3) also correlated with the next session (eg, session 4) PHQ-9 (*r*=–0.02 to –0.46) and GAD-7 (*r*=0.03 to –0.57) changes from session 1. All but 1 correlation coefficient is negative, indicating greater treatment alliance of a session was associated with better treatment response by the next session.

Table 3 shows mixed model results on treatment alliance predicting the next session PHQ-9 and GAD-7 changes from session 1. Participants with higher person-mean treatment alliance showed greater improvements in PHQ-9 (β=–1.13, 95% CI –2.16 to –0.10) and GAD-7 (β=–1.17, 95% CI –2.13 to –0.20). Moreover, the participants in sessions where they reported greater increases in treatment alliance relative to their personal mean predicted greater improvements in PHQ-9 from session 1 (β=–.95, 95% CI –1.90 to –0.00), but not GAD-7. Additionally, the number of completed sessions also significantly predicted improvements in both PHQ-9 and GAD-7.

**Table 3:** Treatment alliance predicting by-session changes in PHQ-9^a^ and GAD-7^b^ scores from session^c^.

Predictor measures	PHQ-9 change, β (95% CI)	*P* value	GAD-7 change, β (95% CI)	*P* value
WAI-Tech^d^ alliance, person mean	–1.13 (–2.16 to –0.10)	.03	–1.17 (–2.13 to –0.20)	.02
WAI-Tech alliance, per-session difference from person mean	–0.95 (–1.90 to –0.00)	.049	–0.55 (–1.45 to 0.35)	.23
Time-variant number of sessions completed	–0.57 (–0.91 to –0.24)	.001	–0.52 (–0.84 to –0.21)	.001

^a^PHQ-9: Patient Health Questionnaire-9.

^b^GAD-7: 7-item Generalized Anxiety Disorder Scale.

^c^Mixed models adjusted for PHQ-9 or GAD-7 score at session 1, sex, race or ethnicity, education, and digital health literacy score.

^d^WAI-Tech: Working Alliance Inventory-Technology Version.

## Discussion

This secondary analysis study explored the associations of objective and subjective user interaction measures as well as treatment response on Lumen, a novel voice-based coach, in a sample of racially and ethnically diverse adults with mild to moderate depression or anxiety or both. The number of conversational breakdowns during each session was relatively low on average, decreasing with sessions, and was not correlated with participant perceptions of workload, user experience, treatment alliance, or their depression or anxiety symptoms. The participants were consistently favorable in their evaluations of the workload and treatment alliance and were neutral to favorable regarding their user experience across the 8 PST sessions with Lumen. User experience was moderately to strongly correlated with treatment alliance across sessions. Both depression and anxiety symptoms improved, with participants on average achieving 3.4 (38%) and 3.2 (31%) reductions in their PHQ-9 and GAD-7 scores, respectively, by the end of the intervention. The treatment alliance predicted the symptom improvements in participants, with a higher mean treatment alliance associated with greater reductions in both depression and anxiety symptoms over the course of the intervention. Moreover, participants, in sessions where they reported higher treatment alliance (relative to their own mean), showed greater reductions in depression, but not anxiety symptoms.

Conversational breakdowns are inevitable and even expected when interacting with current consumer-based voice assistant platforms. Lumen, which was developed on Amazon’s Alexa platform, faced similar, known challenges associated with the platform including those with natural language understanding, tone, and accent, leading to conversational breakdowns [17,18]. Interestingly, conversational breakdowns were not associated with any of the subjective user interaction measures, depression, or anxiety symptoms, suggesting that even when conversational issues occurred, it was generally not an impediment to participant perceptions of their interaction with Lumen or to their treatment response. This is consistent with the finding of a recent experimental study that showed that if a conversational agent offered opportunities for “conversational repair,” participants were more forgiving regarding their user interaction experience [28]. During the design of Lumen [17], extensive testing and design settings were incorporated to create a “resilient” conversational interaction with Lumen to recover from such breakdowns. Lumen is able to provide “conversational repair” by implementing a conversational “failsafe” mechanism such as an ability to repeat and revise conversations. Additionally, over time, Lumen participants faced fewer conversational breakdowns, potentially highlighting how they had adapted to Lumen as a voice-based coach and learned to avoid breakdowns.

Treatment alliance appeared to be the primary user interaction measure that correlated with both depression and anxiety symptoms. The strength of the correlations between treatment alliance and outcomes reported in this study is similar to, or even higher than, that for face-to-face (*r*=0.278) and internet-based psychotherapies (*r*=0.252-0.275) reported in previous studies [29-31]. Also, importantly, this study suggests that both participants who have higher mean treatment alliance and those who experience higher treatment alliance (relative to their mean) during an intervention session are more likely to respond to the treatment.

Even though several communication and sensory modalities (eg, nonverbal behaviors) cannot be used in digital therapies that are limited to voice-based interactions, treatment alliance is an important driver for treatment response for this new delivery modality [30]. The treatment alliance of Lumen may have helped in achieving treatment response, even with the breakdowns in communication. In fact, a previous study reported multiple benefits to this digital modality, including a high level of comfort and openness, and less experience of perceived shame or judgment [32]. Furthermore, voice-based psychotherapy has considerable potential for practice and dissemination in a postpandemic future. Many of the barriers to psychotherapeutic treatment for depression and anxiety can be overcome by this modern information and communication media because it can provide accessibility, flexibility, comfort with disclosure, and cost-related advantages [17].

Treatment alliance could be assessed regularly (eg, at the end of each session) in digital interventions as it can provide real-time insight into treatment outcomes. Any issues associated with treatment alliance should be addressed immediately to help prevent intervention withdrawal and unsatisfactory treatment progress. For example, if specific items related to treatment goal setting showed room for improvement, the voice-based coach might be refined to confirm the accuracy of goals set by the participants and ask participants to rate the importance of achieving goals and confidence in achieving them; if not important or confident, the voice-based coach may ask participants to take additional steps to reflect and refine their goals and to make and execute realistic action plans. This can help the voice-based coach and participants reach a mutual agreement. However, more research is needed to explore how to capture treatment alliance features within a digital environment. In this study, we used the WAI-Tech survey, which kept the same subscales (task, bond, and goal) as the original WAI, but was adapted for digital interventions by rewording the items and omitting human elements [16]. Recent investigations suggested that additional themes (eg, availability and interactivity) might also help account for the complexity of treatment alliance in a digital environment. Qualitative interviews and survey research can help to develop validated and practical questionnaires of digital treatment alliance that are easy to administer for monitoring treatment alliance over the course of a digital intervention.

This study has several strengths. First, the sample is racially and ethnically diverse. Current mental health resources are often limited and underused, especially among these groups. This study provided promising results on the relationship between user interactions and treatment responses in this underserved, mostly minority sample, even though the small sample size precludes subgroup analysis. Second, to the best of our knowledge, Lumen is one of the first, voice-based coach applications for delivering behavioral therapy to treat mild to moderate depression and anxiety. Leveraging longitudinal data on intervention participants from the pilot RCT, this study assessed the repeated user interactions, both objectively and subjectively, with the digital platform through voice input whereby completing a structured 8-session PST program over 3 months. Third, findings from this longitudinal design supported the important role of treatment alliance in predicting treatment outcomes of this novel digital psychotherapy. These strengths address research gaps noted in the previous work [29].

Several limitations are also worth noting. First, the study was based on a small sample of Lumen intervention participants (N=42) in an RCT, limiting the generalizability of the findings. Second, due to its exploratory nature, the study only investigated the relationship between overall scales of task-related workload, user experience, and treatment alliance. The differentiation in the subscales can be investigated in future research. For example, it is hypothesized that task and goal subscales of treatment alliance have higher relations with treatment outcomes than the bond subscale [8]. Whether this hypothesis holds true in digital psychotherapy such as Lumen can be investigated in future work. Third, this study used the existing validated user interaction measures. Among them, only WAI-Tech was specifically adapted for digital interventions. Different therapists (eg, human vs AI coach) may provide different user experiences. More user interaction measures need to be developed for AI tools in future research. Finally, multiple correlation analyses were conducted to investigate bivariate associations among 4 user interaction measures and 2 symptom outcomes across 8 intervention sessions. We opted to not adjust for multiple comparisons due to the exploratory nature of the study. Instead, we focused on moderate or stronger correlation and consistent findings. However, caution in data interpretation is still warranted due to multiple comparisons.

In conclusion, conversational breakdowns were not associated with subjective user interaction measures or treatment responses of this voice-based PST coach in a sample of racially and ethnically diverse adults. Over the course of the intervention, participants exhibited a decreasing trend in conversational breakdowns and reported favorable user interactions. Higher individual mean treatment alliance predicted greater improvements in depression and anxiety, while higher session-based differences from individual mean predicted greater improvements in depression.

## Appendix

Multimedia Appendix 1 Correlations of user interaction with next session Patient Health Questionnaire-9 (PHQ-9) and 7-item Generalized Anxiety Disorder Scale (GAD-7) changes from session 1.

## Abbreviations

AI: artificial intelligence
GAD-7: 7-item Generalized Anxiety Disorder Scale
NASA: National Aeronautics and Space Administration
PHQ-9: Patient Health Questionnaire-9
PST: problem-solving treatment
RCT: randomized clinical trial
REDCap: Research Electronic Data Capture
TLX: Task Load Index
UEQ-S: User Experience Questionnaire Short Version
UIC: University of Illinois Chicago
WAI-Tech: Working Alliance Inventory-Technology Version
