# Supplementary material for: Association Between User Interaction and Treatment Response of a Voice-Based Coach for Treating Depression and Anxiety: Secondary Analysis of a Pilot Randomized Controlled Trial
Source: JMIR Hum Factors. 2023 Nov 6;10:e49715. doi: 10.2196/49715 (PMC10660207; doi:10.2196/49715)
Supplement: Multimedia Appendix 1 [file humanfactors_v10i1e49715_app1.docx]

**Episode**^b^

| **Correlation** | **Episode 1** | **Episode 2** | **Episode 3** | **Episode 4** | **Episode 5** | **Episode 6** | **Episode 7** |
| --- | --- | --- | --- | --- | --- | --- | --- |
|  | **(n=33)** | **(n=29)** | **(n=24)** | **(n=24)** | **(n=22)** | **(n=27)** | **(n=23)** |
| Breakdown- PHQ-9 change | 0.06 | -0.14 | -0.02 | -0.10 | -0.34 | 0.16 | 0.22 |
| NASA TLX- PHQ-9 change | 0.22 | -0.06 | -0.03 | -0.01 | 0.07 | 0.21 | 0.13 |
| UEQ-S- PHQ-9 change | 0.01 | 0.01 | -0.32 | -0.14 | -0.16 | -0.40* | -0.35 |
| WAI-Tech alliance-PHQ-9 change | -0.02 | -0.11 | -0.29 | -0.31 | -0.37 | -0.46* | -0.46* |
|  | | | | | | | |
| Breakdown- GAD-7 change | 0.18 | -0.06 | 0.02 | -0.11 | 0.01 | 0.32 | 0.18 |
| NASA TLX- GAD-7 change | -0.04 | 0.14 | -0.09 | -0.03 | 0.08 | 0.27 | 0.01 |
| UEQ-S- GAD-7 change | -0.15 | -0.32 | -0.36 | -0.34 | -0.53* | -0.35 | -0.23 |
| WAI-Tech alliance-GAD-7 change | 0.03 | -0.33 | -0.52* | -0.57* | -0.48* | -0.41* | -0.24 |

**Appendix 1. Correlations of user interaction with next session PHQ-9 and GAD-7 changes from Session 1**^a^

* Moderate or stronger correlation r≥0.4 or r≤-0.4.

Abbreviations: GAD-7: Generalized Anxiety Disorder-7; NASA TLX: National Aeronautics and Space Administration Task Load Index; PHQ-9: Patient Health Questionnaire-9; UEQ-S: User Experience Questionnaire Short Version; WAI-Tech: Working Alliance Inventory–Technology Version.

^a^ Bivariate Pearson’s correlations were conducted. Each row provides the bivariate Pearson’s correlation coefficients between a pair of user interaction measure and treatment response across 7 episodes.

^b^ Episode (eg, Episode 1) is defined as a pair of user interaction measure completed after a session (eg, Session 1) and PHQ-9 and GAD-7 completed before the next session (eg, Session 2).

| r |  |  |  |  |  |  |
| --- | --- | --- | --- | --- | --- | --- |
|  | -0.6 |  |  | 0 |  | 0.4 |
